# Heat Exhaustion and Heat Stroke Among Active Component Members of the U.S. Armed Forces, 2020–2024

**Published:** 2025-06-20

**Authors:** Alexis L. Maule, Katherine S. Kotas, Kiara D. Scatliffe-Carrion, John F. Ambrose

**Affiliations:** Disease Epidemiology Program, Defense Centers for Public Health–Aberdeen, Defense Health Agency, U.S. Department of Defense, Aberdeen Proving Ground, MD

## Abstract

In 2024, the crude incidence rates of heat stroke and heat exhaustion were 36.4 and 183.9 cases per 100,000 person-years, respectively. After a period of decline in rates of incident heat stroke from 2020 through 2023, during the 2024 surveillance period an increase was observed. When considering only heat exhaustion, incident rates increased each year during the 5-year surveillance period, from 2020 through 2024. In 2024, higher rates of heat stroke were observed among male service members, when compared to their female counterparts, as well as among non-Hispanic White service members compared to service members of other races and ethnicities. Female service members and non-Hispanic Black service members experienced higher rates of heat exhaustion than their male counterparts and service members of other races and ethnicities, respectively. Heat illness rates were also higher among those younger than age 20 years, Marine Corps and Army service members, and recruit trainees. To mitigate the personal and organizational impacts of heat illness, leaders, training cadres, and supporting medical and safety personnel must inform both their subordinate and supported service members of heat illness risks, preventive measures, early signs and symptoms of illness, and appropriate interventions.

Military personnel in the U.S. Armed Forces are required to train and operate at high physical intensity in environments with potentially extreme conditions to maintain force readiness and perform mission essential activities. One occupational hazard of intense physical exertion combined, in some cases, with hot temperatures and humid environments is the occurrence of heat illness. Heat illness refers to a group of disorders that result from a disruption of thermoregulation due to heat stress caused by high energy expenditure (i.e., metabolic heat production), environmental heat exposure, or a combination of both factors. ^1 - 4^

While temperature and humidity are well recognized environmental risk factors for heat illness, there are individual, organizational, and occupational risk factors that influence heat illness occurrence. Individual factors include lack of acclimatization, physical fitness levels, pre-existing or recent viral illness, body composition, and motivation to excel. ^5 - 6^ Organizational factors include type of activity, training intensity and duration, and training schedules. These risk factors do not work independently of each other, there is literature to suggest that risk factors interact to increase risk of heat illness, making it essential that military leaders and service members recognize the full spectrum of factors at play in a training or operational environment. ^7^ For example, metabolic heat production increases during prolonged engagement in strenuous physical activity, and additional exposure to environmental heat stress elevates core and skin temperatures. ^2 , 3 , 8^

**What are the new findings?**

In 2024, the crude annual incidence rate of heat stroke increased 16.5%, following a 4-year decrease of 10.8% from 2020 to 2023. All services, apart from the Space Force and Coast Guard, had a higher rate of heat stroke in 2024 than in 2023. The crude annual incidence rate of heat exhaustion increased 52.3% from 2020 to 2024, with incremental increases annually. Increased rates of heat exhaustion in 2024 from 2023 were observed in the Army, Marine Corps, and Coast Guard.

**What is the impact on readiness and force health protection?**

The most serious types of heat illnesses, heat exhaustion and heat stroke, are occupational hazards associated with many of the military's training and operational environments, posing potential risks for force health protection. Heat exhaustion and heat stroke can typically be prevented by accurate situational awareness, appropriate risk management strategies, and effective countermeasures. Units that fail to implement heat illness mitigation measures risk impeding or interrupting training programs, resulting in otherwise preventable reductions in operational tempo or critical mission failure due to lost personnel and resources.

While identifying high-risk service members is critical in preventing heat illness and reducing morbidity due to heat illnesses, heat illness mitigation strategies can be layered using a risk management model. ^9^ For achieving the goal of hazard reduction, progressive training, heat acclimatization, and ensuring proper hydration, electrolyte replacement, and nutrition prior to training are strategies that can prepare service members for training and operating in high heat environments. ^3 , 10^ Risk mitigation strategies that can be applied during training events include implementing work and rest guidelines, modifying clothing and uniform standards, adopting self- or group-pacing during high-risk events (e.g., ruck marches), modifying schedules or activities, and providing cooling measures (e.g., arm immersion cooling or microclimate cooling). ^9 - 11^ Further, early detection reduces morbidity and severity of heat illness and can be achieved by educating service members and leadership on the signs and symptoms of heat illness, incorporating physiological monitoring, managing outliers during training (i.e., establishing minimum or maximum pacing), and removing individuals from high-risk events. Finally, effective management of heat casualties includes applying proper cooling techniques (e.g., cold water immersion, iced sheets). ^6 , 9 , 11^

Heat illness occurs within a continuum of severity, from less severe (e.g., heat cramps, rash, edema, syncope), to heat exhaustion, followed by potentially life-threatening heat stroke. Heat exhaustion and heat stroke are reportable medical events (RMEs) in the Military Health System (MHS). All heat casualties that require medical intervention or result in change of duty status must be reported. ^12^ During or immediately following a period of physical exertion or heat exposure, specific signs and symptoms that characterize heat illnesses allow initial recognition of their occurrence in the field, and subsequent identification or diagnosis of a heat illness that should be reported.

Common signs and symptoms of heat exhaustion include weakness, muscle cramps, headache, dizziness, nausea or vomiting, tachycardia, and short-term physical collapse or debilitation. Heat exhaustion is often characterized by evidence of elevated core body temperature (not greater than 104 °F, 40 °C) with no significant central nervous system dysfunction. If any central nervous system dysfunction develops (e.g., dizziness, confusion, headache), it should be mild and rapidly resolve with rest and cooling measures, otherwise the individual may be experiencing heat stroke. ^5 , 8 , 13 - 14^

Heat stroke is a debilitating and potentially life-threatening condition most frequently characterized by evidence of severe hyperthermia (greater than or equal to 104 °F, 40 °C) and central nervous system dysfunction (e.g., change in mental status, delirium, stupor, loss of consciousness, coma). ^5 , 8 , 13 , 15^ Onset of heat stroke should prompt aggressive intervention featuring rapid cooling (e.g., iced sheets). The literature on heat stroke management indicates consensus on prioritizing cooling over transport for further medical attention. ^9 , 15 - 17^ Cooling is prioritized because, clinically, severity of end-organ damage and increased possibility of mortality are directly related to the degree and duration of hyperthermia. ^8 , 11 , 17^ End-organ damage is most frequently observed in the liver, kidneys, cardiac, and skeletal muscle. ^8 , 15 - 16 , 18^

Ongoing surveillance of heat illnesses is necessary to determine if prevention guidelines and countermeasures are working, in addition to identifying high-risk groups and activities that may lead to heat illness. Since 2001 *MSMR* has published regular updates on the incidence of heat illness among U.S. active component service members (ACSMs). This update presents summaries of heat stroke and heat exhaustion case counts, incidence rates, and locations from 2020 through 2024.

## Methods

The surveillance population for this analysis includes all individuals who served in the active component of the Army, Navy, Marine Corps, Air Force, Space Force, or Coast Guard at any time during the surveillance period of January 1, 2020 through December 31, 2024. Space Force data are only complete for 2023 and 2024.

All data used to determine incident heat illness diagnoses were derived from 4 sources: MHS Management, Analysis and Reporting Tool (M2); Defense Medical Surveillance System (DMSS); Disease Reporting System internet (DRSi); and Theater Medical Data Store (TMDS). Heat illness cases were identified using specific diagnostic codes from the ambulatory care encounters and hospitalizations of ACSMs in fixed military and civilian (if reimbursed through the MHS) hospitals and clinics worldwide. In addition to medical encounter data, heat illness medical event reports were identified in DRSi, including information on hospitalization status (i.e., yes or no). If a heat illness was reported in DRSi, but not found in the medical record, the case was still counted. For example, an individual could be treated in the field by a medic for a mild or non-life-threatening heat illness without a recorded medical encounter, but the case is deemed a reportable heat exhaustion because of symptoms observed in the field.

In this update, a case of heat illness was defined as an individual with 1) a hospitalization or outpatient medical encounter record or outpatient medical encounter record with a primary (first-listed) or secondary (second-listed) diagnosis of heat stroke (International Classification of Diseases, 9th Revision [ICD-9]: 992.0; International Classification of Diseases, 10th Revision [ICD-10]: T67.0*) or heat exhaustion (ICD-9: 992.3 – 992.5; ICD-10: T67.3* – T67.5*) or 2) a RME record of heat exhaustion or heat stroke ^19^ ; asterisks denote that all subsequent digits or characters noted in that diagnostic code were included in the identification of ICD-10 codes (e.g., T67.3XXA).

An individual was considered a case of heat illness only once per calendar year. If a service member had diagnoses for both heat stroke and heat exhaustion during a given year, the more severe diagnosis (i.e., heat stroke) was selected. If a service member had inpatient and outpatient encounter for heat stroke or heat exhaustion, the inpatient encounter was prioritized over the outpatient visit, when identifying hospitalized cases. Within a calendar year, if an individual had a diagnostic code that denoted a subsequent encounter (i.e., ICD-10 seventh digit ‘D’) or an encounter for sequelae (i.e., ICD-10 seventh digit ‘S’), but had no diagnostic codes indicating an initial visit (i.e., ICD-10 seventh digit ‘A’), the case was removed to avoid over-estimating heat illness cases by including those receiving follow-up care.

For health surveillance purposes, recruit trainees were identified as ACSMs assigned to service-specific training locations and basic training periods, using an algorithm based on age, rank, and time in service. Recruit trainees were considered a separate enlisted service member category in heat illness summaries by military grade. In summaries of heat illness by location, the Defense Medical Information System Identifier (DMIS ID) was utilized to determine installation or geographic location of diagnosis and medical treatment.

In-theater diagnoses of heat illness, within the U.S. Central Command (CENTCOM) area of responsibility (AOR), were identified from medical records of deployed service members whose health care encounters were documented in TMDS. Those encounters were analyzed separately, and the same case-defining criteria and incidence rules described previously were applied.

Incidence rates were calculated as incident cases of heat illness per 100,000 ACSM person-years (p-yrs). Percent change in incidence was calculated using unrounded rates. Because reporting of heat exhaustion and heat stroke cases is required, the proportion of outpatient and inpatient cases with a report in DRSi was also calculated. ^12^

## Results

In 2024, 471 cases of heat stroke were reported throughout the MHS, resulting in a crude incidence rate of 36.4 cases per 100,000 p-yrs **(Table 1)** . Subgroup-specific incidence rates of heat stroke were highest among men, those younger than age 20 years, non-Hispanic White service members, Marine Corps and Army personnel, recruit trainees, as well as those in combatspecific occupations. The incidence rate of heat stroke among recruit trainees was 3.3 and 3.5 times higher than other enlisted service members and officers, respectively.

**TABLE 1.** Incident Cases ^a^ and Incidence Rates ^b^ of Heat Illness, Active Component, U.S. Armed Forces, 2024

	Heat Stroke		Heat Exhaustion		Total Heat Illness Diagnoses	
	No.	Rate ^b^	No.	Rate ^b^	No.	Rate ^b^
Total	471	36.4	2,380	183.9	2,851	220.3
Sex						
Male	414	38.9	1,885	177.2	2,299	216.1
Female	57	24.8	495	215.1	552	239.8
Age group, y						
<20	86	104.9	624	761.2	710	866.1
20–24	196	50.6	1,020	263.1	1,216	313.7
25–29	130	42.7	406	133.4	536	176.1
30–34	37	17.1	182	84.2	219	101.3
35–39	15	9.0	97	57.9	112	66.8
40+	7	5.1	51	37.5	58	42.6
Race and ethnicity						
White, non-Hispanic	305	45.8	1,454	218.5	1,759	264.3
Black, non-Hispanic	80	38.1	495	235.8	575	273.9
Hispanic	24	9.3	141	54.6	165	63.8
Other / unknown ^c^	62	38.8	290	181.3	352	220.0
Service branch						
Army	246	55.8	1,207	273.9	1,453	329.7
Navy	42	13.0	201	62.0	243	75.0
Air Force	39	12.5	297	95.3	336	107.9
Marine Corps	143	84.7	655	388.2	798	472.9
Space Force	0	0.0	4	43.1	4	43.1
Coast Guard	1	2.5	16	40.5	17	43.0
Military status						
Recruit trainee	27	116.6	553	2,388.9	580	2,505.5
Enlisted	363	35.3	1,643	159.8	2,006	195.1
Officer	81	33.4	184	75.8	265	109.2
Military occupation						
Combat-specific ^d^	156	96.5	485	300.1	641	296.6
Motor transport	8	19.0	66	156.6	74	175.6
Pilot / air crew	7	15.5	13	28.9	20	44.4
Repair / engineering	34	9.4	148	41.1	182	50.6
Communications / intelligence	10	3.6	30	10.8	40	14.4
Health care	24	22.8	96	91.1	120	113.9
Other / unknown	232	76.8	1,542	510.8	1,774	587.6

Abbreviations: No., number; y, years.

a One case per person per calendar year.

b Rate per 100,000 person-years.

c Includes those of American Indian / Alaskan Native, Asian, Native Hawaiian / Pacific Islander, and unknown race or ethnicity.

d Infantry / artillery / combat engineering / armor.

**FIGURE 1.**
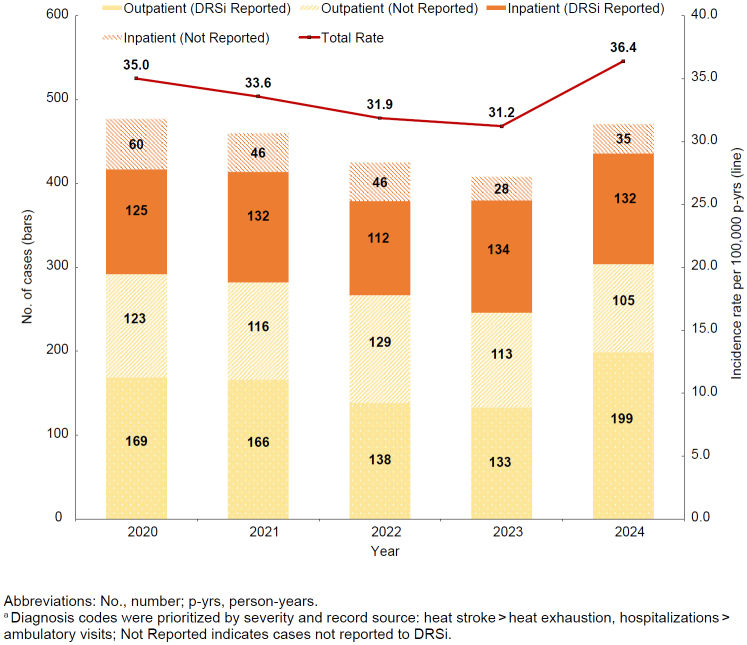
Incident Cases ^a^ and Incidence Rate of Heat Stroke, by Encounter Type and Year of Diagnosis, Active Component, U.S. Armed Forces, 2020–2024

After decreasing for 4 years, in 2024 the crude annual incidence of heat stroke increased 16.5% **(Figure 1)** . Incidence rates of heat stroke increased in 2024 among service members in the Air Force (95.3%), Marine Corps (25.6%), Navy (17.8%), and Army (6.4%) **(Table 2)** . Meanwhile, the proportion of heat stroke cases that were hospitalized decreased, to 35.5% in 2024, from 39.7% in 2023 **(Figure 1)** . Of all inpatient heat stroke cases from 2020 through 2024, 74.7% were reported to DRSi, while over half (57.9%) of outpatient heat stroke cases had a medical event report in DRSi.

**TABLE 2.** Annual Incident Cases ^a^ and Incidence Rates ^b^ of Heat Illness, by Service, Active Component, U.S. Armed Forces, 2020–2024

	Army		Navy		Air Force		Marine Corps		Space Force		Coast Guard	
	No.	Rate ^b^	No.	Rate ^b^	No.	Rate ^b^	No.	Rate ^b^	No.	Rate ^b^	No.	Rate ^b^
Heat exhaustion												
2020	899	189.2	135	40.1	130	39.6	473	259.8	—	—	9	22.2
2021	1,034	216.0	147	43.0	183	55.6	496	276.5	—	—	5	12.4
2022	1,036	224.9	190	56.2	237	73.8	555	319.1	—	—	19	47.9
2023	1,190	265.5	207	63.3	363	115.3	488	288.9	7	81.9	6	15.5
2024	1,207	273.9	201	62.0	297	95.3	655	388.2	4	43.1	16	40.5
Heat stroke												
2020	284	59.8	24	7.1	17	5.2	152	83.5	—	—	0	0.0
2021	297	62.1	25	7.3	27	8.2	111	61.9	—	—	0	0.0
2022	226	49.1	31	9.2	32	10.0	135	77.6	—	—	1	2.5
2023	235	52.4	36	11.0	20	6.4	114	67.5	0	0.0	3	7.7
2024	246	55.8	42	13.0	39	12.5	143	84.7	0	0.0	1	2.5
Total heat illness diagnoses												
2020	1,183	249.0	159	47.3	147	44.7	625	343.3	—	—	9	22.2
2021	1,331	278.1	172	50.3	210	63.8	607	338.4	—	—	5	12.4
2022	1,262	274.0	221	65.4	269	83.7	690	396.7	—	—	20	50.4
2023	1,425	318.0	243	74.3	383	121.7	602	356.4	7	81.9	9	23.2
2024	1,453	329.7	243	75.0	336	107.9	798	472.9	4	43.1	17	43.0

Abbreviations: No., number; y, years.

a One case per person per calendar year.

b Rate per 100,000 person-years.

The 2,380 cases of heat exhaustion in 2024 correspond to a crude incidence rate of 183.9 cases per 100,000 p-yrs **(Table 1)** . As with heat stroke, higher rates of heat exhaustion were noted for service members younger than age 20 years, Marine Corps and Army personnel, and recruit trainees. Unlike heat stroke, however, the rate of heat exhaustion was higher among women (21.4% higher compared to men) and non-Hispanic Black service members (7.9% higher compared to non-Hispanic White service members). The incidence rate of heat exhaustion among recruit trainees was 14.9 and 31.5 times higher than other enlisted service members and officers, respectively.

**FIGURE 2.**
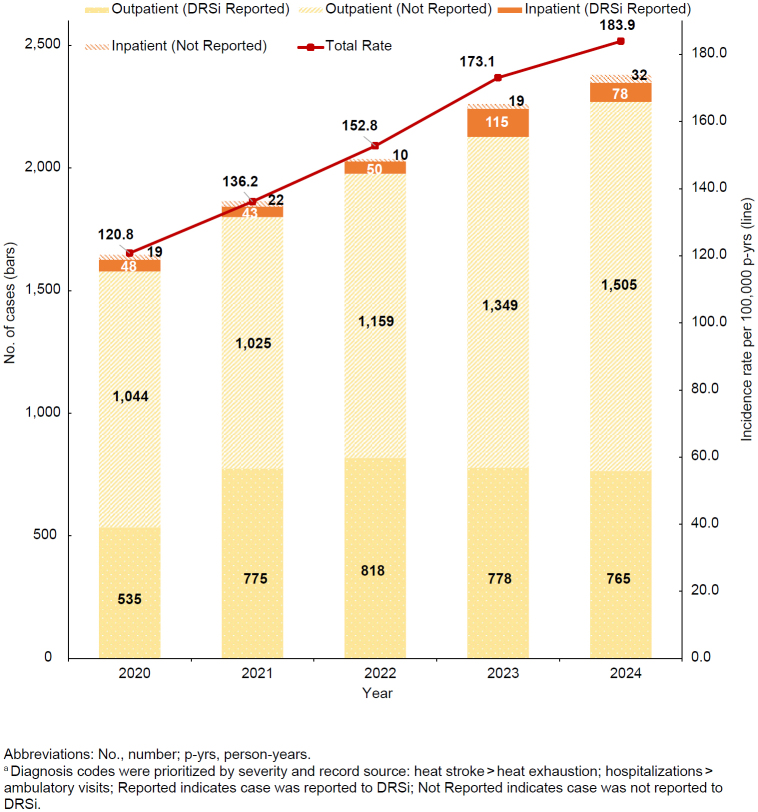
Incident Cases ^a^ and Incidence Rates of Heat Exhaustion, by Encounter Type and Year of Diagnosis, Active Component, U.S. Armed Forces, 2020–2024

The crude annual incidence rate of heat exhaustion increased each year of the 5-year surveillance period **(Figure 2)** , with a 52.3% increase from 2020 through 2024—including a 6.3% increase from 2023 to 2024. Service-specific increases in incidence rates of heat exhaustion were observed in 2024 among Marine Corps (34.3%) and Army personnel (3.1%) from the rates observed in 2023 **(Table 2)** . The proportion of heat exhaustion cases that were hospitalized in the U.S. Armed Forces decreased, however, from 2023 (5.9%) to 2024 (4.6%) **(Figure 2)** .

Over three-quarters (76.6%) of inpatient heat exhaustion cases were reported in DRSi from 2020 to 2024, while only 37.6% of outpatient heat exhaustion cases had a medical event report in DRSi.

### Heat illnesses by location

During the 5-year surveillance period, 12,430 heat illness cases were diagnosed at more than 300 military installations and geographic areas worldwide **(Table 3)** . Only 6.9% of those heat illness cases occurred outside the U.S., including 344 in Okinawa, Japan. From 2020 to 2024, 22 locations reported at least 100 cases of heat illness, and those 22 locations accounted for over three-quarters (77.4%) of all active component cases. Three Army installations (Fort Benning, GA; Fort Bragg, NC; Fort Campbell, KY), 2 Marine Corps bases (MCB Camp Lejeune / Cherry Point, NC, MCRD Parris Island / Beaufort, SC), and 1 Joint Base (JB San Antonio, TX) accounted for 44% of the total heat illnesses during the surveillance period. Of the 22 locations with at least 100 cases of heat illness, 15 are in the southern U.S.

**TABLE 3.** Heat Injury Events ^a^ by Location of Diagnosis or Report with at Least 100 Cases During the Period, Active Component, U.S. Armed Forces, 2020–2024

Location of Diagnosis	No.	% Total
Fort Benning, GA	1,839	14.8
MCB Camp Lejeune / Cherry Point, NC	894	7.2
Fort Bragg, NC	833	6.7
JB San Antonio, TX	656	5.3
MCRD Parris Island / Beaufort, SC	642	5.2
Fort Campbell, KY	606	4.9
Fort Johnson, LA	475	3.8
MCRD San Diego / NB San Diego	473	3.8
Fort Cavazos, TX	425	3.4
Okinawa, Japan	344	2.8
MCB Camp Pendleton, CA	333	2.7
MCB Quantico, VA	330	2.7
Fort Jackson, SC	318	2.6
Fort Sill, OK	287	2.3
Fort Irwin, CA	197	1.6
Twentynine Palms, CA	183	1.5
Fort Shafter, HI	159	1.2
Fort Stewart, GA	155	1.2
Fort Leonard Wood, MO	139	1.1
Fort Bliss, TX	128	1.0
NAS Pensacola, FL	105	0.8
NSA Annapolis, MD	103	0.8
Outside U.S. ^b^	510	4.1
All other locations	2,296	18.5
Total	12,430	100

Abbreviations: No., number; MCB, Marine Corps Base; MCRD, Marine Corps Recruit Depot; NAS, Naval Air Station; NB, Naval Base; NSA, Naval Support Activity; JB, Joint Base.

a One heat illness per person per year.

b Excluding Okinawa, Japan.

Note: Initial entry recruit training locations include Fort Jackson, Fort Leonard Wood, Fort Benning, Fort Sill, MCRD Parris Island / Beaufort, MCRD San Diego / NB San Diego, and JB San Antonio. Fort Johnson is the Joint Readiness Training Center (JRTC) and Fort Irwin is the National Training Center (NTC).

### Heat illnesses in CENTCOM AOR

**FIGURE 3.**
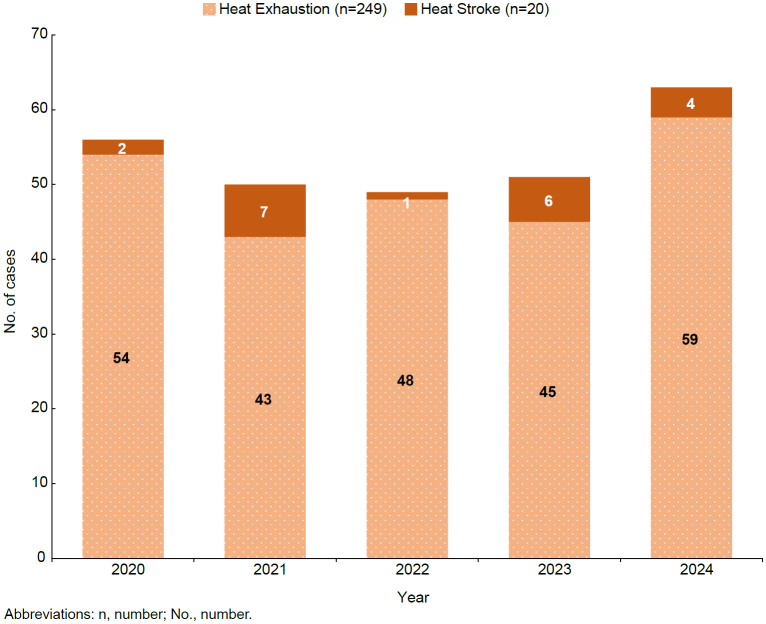
Incident Cases of Heat Illnesses in CENTCOM Area of Responsibility, Active Component, U.S. Armed Forces, 2020–2024

During the 5-year surveillance period, 269 cases of heat illness were reported in the CENTCOM AOR **(Figure 3)** . Of those 269 cases of heat illness, 7.4% (n=20) were heat stroke. Cases of heat illness occurred most frequently among deployed service members who were male (n=188, 69.9%), 20-24 years old (n=133, 49.4%), and in the Navy (n=119, 44.2%) or Air Force (n=75, 27.9%) (data not shown).

## Discussion

During the 5-year surveillance period, the rate of heat stroke decreased annually from 2020 through 2023, but then increased in 2024. The rate of heat exhaustion increased steadily from 2020 to 2024. Between 2023 and 2024, the rate of heat stroke increased 16.5%, and the rate of heat exhaustion increased 6.3%. For both heat stroke and heat exhaustion cases, however, the percentage of hospitalized cases declined from 2023 to 2024. While the reason for the increase in heat illness cases in 2024 is unknown, the decrease in hospitalized cases is indicative of fewer severe cases of heat illness that required higher level inpatient medical care. It is possible that current heat illness prevention guideline emphasis on education about the signs, symptoms, and management of heat casualties leads to earlier and more frequent recognition of what constitutes a heat illness while also preventing more severe heat illness. ^3 , 10 , 20 - 21^

There are limitations to this update that should be considered when interpreting its findings. Although heat illnesses were summarized by the location of diagnosis or report, medical care may not occur at the same location (i.e., installation) as the heat illness event, particularly if the case required a level of care not available locally. To account for locations with medical care redundancy, some installations were combined (e.g., MCB Camp Lejeune/Cherry Point, NC in **Table 3** ); this merging of locations was most prevalent with Marine Corps and Navy locations. In addition, the personnel files from the Defense Manpower Data Center that were utilized to calculate population estimates for the active component as well as the demographic data presented in **Table 1** were unavailable for October through December 2024; the duty statuses of all service members in September 2024 were assumed to be their duty statuses through the end of the calendar year. It is likely that some individuals in the U.S. Armed Forces both joined and left service during those months, and those movements are unaccounted for in the population estimates. Likewise, it is possible some time-varying demographics (e.g., rank) changed for individuals from October through December 2024 compared to September, and those shifts in categories are unaccounted for. In all instances, however, the effect on the rates shown throughout the report should be minimal due to the large population size.

Further, the method used to identify recruit trainees likely resulted in some misclassification of recruit training status. The algorithm did not account for the additional training time in the Army's One Status Unit Training beyond the traditional basic combat training period and does not account for service members who are recycled through training, likely leading to an under-estimation of the incident cases and incidence rates of heat illnesses among recruit trainees. Finally, there was likely incomplete capture of heat illnesses treated in the field during training and deployments, rather than at a fixed military hospital or clinic; this may be particularly true for heat exhaustion cases when symptoms rapidly resolve after a period of rest.

Maintaining regular heat illness surveillance helps identify the magnitude of the impact these conditions have on service member health, training, and force readiness. At the command and unit level, emphasis on evidence-based prevention, mitigation and risk management, with continued education on the signs, symptoms, and early field interventions for heat illness, are crucial steps in reducing the impact of heat illness morbidity on the force. To ensure protection throughout the force, DOD standards, policies, or procedures should determine the prevention, mitigation, and management of heat illnesses.
